# Genome-Wide Identification and Characterization of Warming-Related Genes in *Brassica rapa* ssp. *pekinensis*

**DOI:** 10.3390/ijms19061727

**Published:** 2018-06-11

**Authors:** Hayoung Song, Xiangshu Dong, Hankuil Yi, Ju Young Ahn, Keunho Yun, Myungchul Song, Ching-Tack Han, Yoonkang Hur

**Affiliations:** 1 Department of Biological Sciences, Chungnam National University, Daejeon 34141, Korea; hysong@cnu.ac.kr (H.S.); hankuil.yi@cnu.ac.kr (H.Y.); wnduds357@naver.com (J.Y.A.); keunho0307@gmail.com (K.Y.); 2 School of Agriculture, Yunnan University, Kunming 650091, China; dongxiangshu_123@163.com; 3 Department of Life Science, Sogang University, Seoul 04107, Korea; s000692@sogang.ac.kr (M.S.); cthan@ccs.sogang.ac.kr (C.-T.H.)

**Keywords:** warming, BrHSFA2, BrHSP18.2s, transcriptome, alternative splicing, Kenshin

## Abstract

For sustainable crop cultivation in the face of global warming, it is important to unravel the genetic mechanisms underlying plant adaptation to a warming climate and apply this information to breeding. Thermomorphogenesis and ambient temperature signaling pathways have been well studied in model plants, but little information is available for vegetable crops. Here, we investigated genes responsive to warming conditions from two *Brassica rapa* inbred lines with different geographic origins: subtropical (Kenshin) and temperate (Chiifu). Genes in Gene Ontology categories “response to heat”, “heat acclimation”, “response to light intensity”, “response to oxidative stress”, and “response to temperature stimulus” were upregulated under warming treatment in both lines, but genes involved in “response to auxin stimulus” were upregulated only in Kenshin under both warming and minor-warming conditions. We identified 16 putative high temperature (HT) adaptation-related genes, including 10 heat-shock response genes, 2 transcription factor genes, 1 splicing factor gene, and 3 others. *BrPIF4*, *BrROF2*, and *BrMPSR1* are candidate genes that might function in HT adaptation. Auxin response, alternative splicing of *BrHSFA2*, and heat shock memory appear to be indispensable for HT adaptation in *B. rapa*. These results lay the foundation for molecular breeding and marker development to improve warming tolerance in *B. rapa*.

## 1. Introduction

Global warming poses a serious threat to agriculture, as it threatens crop productivity and food safety worldwide [1,2]. Various strategies have been utilized to facilitate the breeding or engineering of thermotolerant crops, including regulating the expression of heat shock (HS) transcription factor (HSF) and heat shock response (HSR) genes, as well as the use of molecular markers [1,3,4].

In a model plant *Arabidopsis thaliana*, three types of tolerance responses to heat exposure have been identified: basal thermotolerance, acquired thermotolerance, and warming tolerance [5,6,7]. Plants with basal thermotolerance can survive when grown at 21–22 °C (normal growth condition), exposed to 42–45 °C for 0.5–1 h, and examined after 5–7 days. Plants with acquired thermotolerance can survive when grown under normal conditions, transferred to 36–38 °C (moderate heat stress) for 1.5 h (referred to as “priming”), recovered at 21–22 °C for 2 h, subjected to over 45 °C, and examined after 5–7 days. Plants with warming tolerance (as opposed to heat-stress tolerance) survive when grown under normal conditions, subjected to 12 °C for 2 days, and treated with warming conditions (27 °C) for 3 h. The long-term adaptation of plants to warmer growth conditions is thought to induce developmental reprograming. Identifying and applying warming-related genes in *Brassica* crop species poses a major challenge for crop breeding for improved tolerance to global warming.

Several marker genes for thermotolerance responses are currently available [8]. Genes encoding an exportin family protein (*XPO1A*, AT5G17020) and heat shock protein (HSP) 101 (*HSP101*, AT1G74310) are markers for basal thermotolerance. Acquired thermotolerance by priming is divided into two categories: short-term and long-term acquired thermotolerance. *HSP101* expression represents short-term acquired thermotolerance, while long-term acquired thermotolerance is characterized by the expression of several marker genes, including those encoding Rotamase FKBP1/FK506-binding protein 62 (*ROF1/FKBP62*, AT3G25230), ROF2/FKBP65 (AT5G48570), heat HSF factor A2 (*HSFA2*, AT2G26150), HSP101, and heat stress-associated 32 kD (*Hsa32*, AT4G21320). During the long-term acquired thermotolerance response, the expression levels of small HSP genes (*sHSP*s), *HSP70*s, *ROS* genes, and ascorbate peroxidase (*APX*) also increase [7].

Warming treatment does not trigger the expression of HSR genes, but other genes, such as *HSP70* (AT3g12580) [6] and *Phytochrome-Interacting Factor4* (*PIF4*, AT2G43010) [9,10,11], are core components in this process. PIF4 controls morphological acclimation to high temperatures (HT) via auxin [9,12]. Phytochrome B (PhyB) was recently shown to function upstream of PIF4 [13]. Changes in ambient temperatures induce alternative splicing of a large number of genes [14]. Due to increases in global temperatures, the mechanism used by plants to sense small variations in ambient temperatures is becoming an increasing focus of study. It is important to elucidate whether crops that have long been cultivated in regions with different climates, such as Chinese cabbage, have developed similar responses to warming to those found in *Arabidopsis*.

Two inbred Chinese cabbage lines, Chiifu and Kenshin, have different geographic origins: Chiifu originated in temperate regions, whereas Kenshin originated in subtropical and tropical regions. Kenshin has long been used as a breeding stock to develop heat-tolerant *Brassica* species [15,16]. In addition, these two inbred lines show different electrolyte leakage rates in response to HT exposure and different expression of many genes [17]. The long history and intensive breeding of these two Chinese cabbage lines make them promising targets for transcriptome analysis after warming treatment to identify warming-related genes in this crop. These genes could then be used to develop molecular markers and to generate climate-change-resilient *Brassica* crops under global warming conditions. In the current study, we used the Br135K microarray (Version 3) to identify differentially expressed genes (DEGs) upon warming treatment in Chiifu and Kenshin Chinese cabbage, confirmed their expression patterns by qRT-PCR, and further characterized the expression of patterns of several candidate warming-relating genes. The results of this study lay the foundation for breeding Chinese cabbage lines with improved tolerance to warming conditions.

## 2. Results

### 2.1. Transcriptome Analysis of Plants under Warming, Minor-Warming, and Low-Temperature (LT) Conditions Using the Br135K Microarray

**Figure 1:**
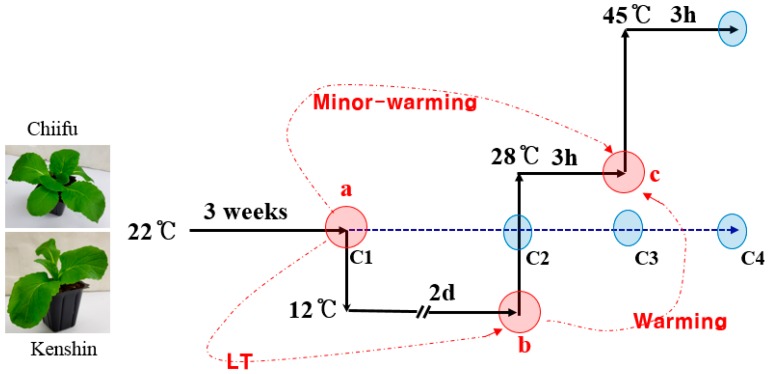
Temperature treatment and sampling schedule. Collection times are indicated by circles. Red circles (**a**–**c**) represent sampling times for the microarray experiments as well as qRT-PCR analysis. Blue circles indicate that the collected samples were only used for qRT-PCR. Shoots from five individual plants were sampled and frozen in liquid nitrogen. Treatments were as follows: (**a**) (22 °C) to (**b**) (12 °C); low-temperature (LT) conditions; (**a**) (22 °C) to (**c**) (28 °C), minor-warming conditions; and (**b**) (12 °C) to (**c**) (28 °C), warming conditions.

To identify putative warming-related (or HT adaptation-related) genes, we carried out Br135K microarray analysis of samples from two inbred lines, Chiifu and Kenshin, under three conditions (22 °C, 12 °C, and 12 ⟶ 28 °C) (Figure 1). The experiments were repeated twice; the mean values are summarized in Table S2. The microarray data have been deposited in “NCBI (https://www.ncbi.nlm.nih.gov/)” with [geo] GSE113637. Among the 41,173 genes deposited on the Br135K microarray, 14,222 (35%) showed probe intensity (PI) values <500 in all samples, whereas 26,951 (65%) showed PI values >500 in at least one sample. Of these 26,951 genes, 2104 had no *Arabidopsis* counterpart, i.e., NA (nonannotated genes). We subjected these 26,951 genes to further analysis because genes with a PI value of 500 (cutoff value) can easily be examined using standard RT-PCR. Responsive genes (i.e., DEGs) were defined as having at least a 2-fold change (cutoff value) in expression between comparative conditions. As shown in Figure 1, three types of comparisons were made: warming (samples treated for 2 days at 12 °C vs. samples after 3 h exposure to 28 °C), minor-warming (samples grown at 22 °C [control] vs. samples after 3 h exposure to 28 °C), and low-temperature (LT) treatment (samples grown at 22 °C vs. samples treated for 2 days at 12 °C).

#### 2.1.1. Warming-Responsive Genes

**Figure 2:**
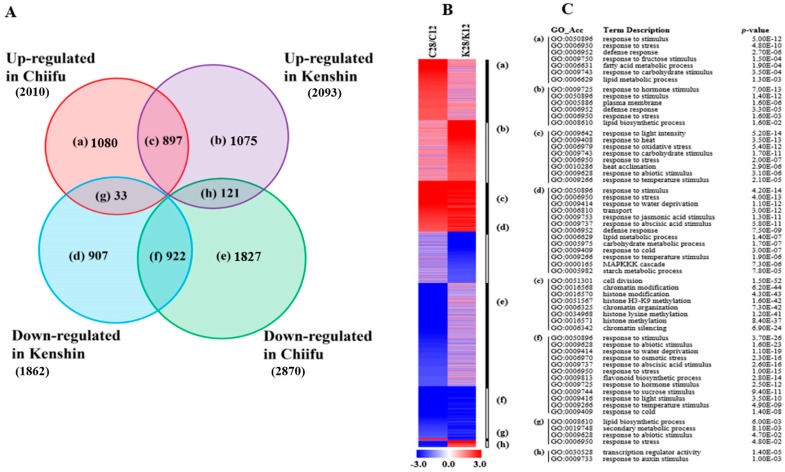
Analysis of warming-responsive genes from two contrasting inbred lines, Chiifu and Kenshin. (**A**) Venn diagram of DEGs with over 2-fold differences in expression; a–h indicate the groups of genes in each category; (**B**) Heatmap of DEGs in each group based on fold change; (**C**) Gene Ontology (GO) enrichment analysis of DEGs in each group, with *p* values obtained using the agriGO tool (http://bioinfo.cau.edu.cn/agriGO/index.php). C and K of Figure 2B indicate Chiifu and Kenshin, respectively.

We identified 6862 warming-responsive genes. Similar numbers of these genes were upregulated in Chiifu and Kenshin, but more were downregulated in Chiifu (Figure 2A; Table S3). Genes in the Gene Ontology (GO) biological process categories “response to heat”, “heat acclimation”, “response to light intensity”, “response to oxidative stress”, and “response to temperature stimulus” were enriched among upregulated genes in both lines (group (c) in Figure 2B,C). The categories “response to hormone stimulus”, “plasma membrane”, “defense response”, and “lipid biosynthetic process” were enriched among genes that were specifically upregulated in Kenshin (group (b) in Figure 2B,C). Genes in the categories “response to water deprivation”, “response to osmotic stress”, “response to sucrose stimulus”, and “response to cold” were enriched among downregulated genes in both lines (group (f) in Figure 2B,C). Genes in two categories, “transcription regulatory activity” and “response to auxin stimulus”, were upregulated in Kenshin but downregulated in Chiifu under warming conditions (group (h) in Figure 2B,C).

#### 2.1.2. Minor Warming-Responsive Genes

**Figure 3:**
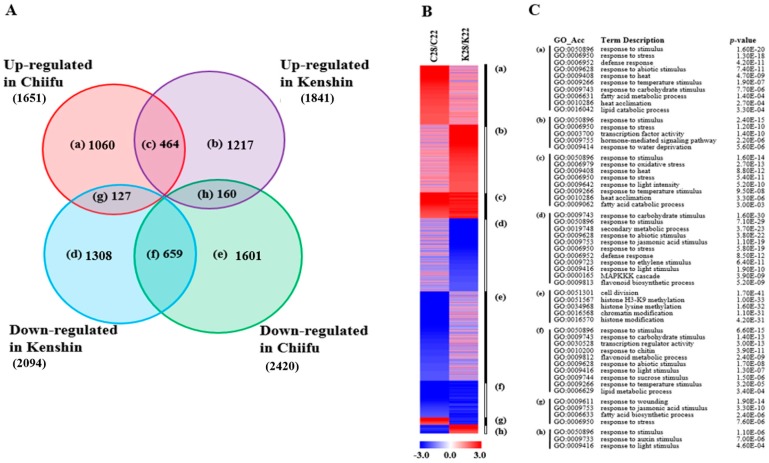
Analysis of minor warming-responsive genes from two contrasting inbred lines, Chiifu and Kenshin. (**A**) Venn diagram of DEGs with over 2-fold differences in expression; a–h indicate the groups of genes in each category; (**B**) Heatmap of DEGs in each group based on fold change; (**C**) GO enrichment analysis of DEGs in each group, with *p* values obtained using the agriGO tool (http://bioinfo.cau.edu.cn/agriGO/index.php). C and K of Figure 3B indicate Chiifu and Kenshin, respectively.

We identified 6596 minor warming-responsive genes; more of these genes were upregulated in Kenshin and downregulated in Chiifu (Figure 3A; Table S4). Upregulated genes in both lines were enriched in similar categories to those of warming-responsive genes, such as “response to heat”, “heat acclimation”, “response to light intensity”, “response to oxidative stress”, and “response to temperature stimulus” (group (c) in Figure 3B,C). Genes upregulated specifically in Kenshin were enriched in the categories “response to stimulus”, “response to stress”, “transcription factor activity”, and “response to water deprivation” (group (b) in Figure 3B,C). Genes in three categories, “response to stimulus”, “response to light stimulus”, and “response to auxin stimulus”, were upregulated in Kenshin but downregulated in Chiifu in response to minor-warming conditions (group (h) in Figure 3B,C). The category “response to auxin stimulus” was enriched among genes upregulated in Kenshin under both warming and minor-warming conditions, suggesting that the auxin response might contribute to heat tolerance in Kenshin.

#### 2.1.3. LT-Responsive Genes

**Figure 4:**
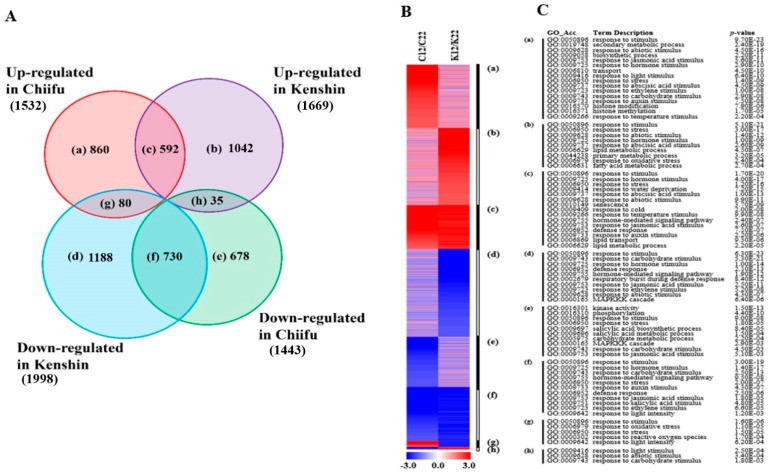
Analysis of LT-responsive genes from two contrasting inbred lines, Chiifu and Kenshin. (**A**) Venn diagram of DEGs with over 2-fold differences in expression; a–h indicate the groups of genes in each category; (**B**) Heatmap of DEGs in each group based on fold change; (**C**) GO enrichment analysis of DEGs in each group, with *p* values obtained using the agriGO tool (http://bioinfo.cau.edu.cn/agriGO/index.php). C and K of Figure 4B indicate Chiifu and Kenshin, respectively.

The identification of LT-responsive genes was not the main objective of this study, but associations between cold- and heat-stress signaling through calcium signaling, ROS signaling, and protein degradation have been reported [18,19,20], and changes in DNA methylation were also shown to be involved in heat tolerance [21]. In addition, we wanted to examine the possible association between LT treatment and the warming response. We identified 5205 LT-responsive genes, for which more genes were upregulated in Kenshin and downregulated in Chiifu (Figure 4; Table S5), implying that Kenshin is more sensitive to LT exposure than Chiifu. Many GO categories were identified as enriched among upregulated genes in both lines, including “response to cold”, “response to stress”, and “lipid transport and metabolic process”. Although similar GO categories were enriched among LT-responsive genes and genes in the two categories mentioned above (warming and minor warming), the expression of warming- and minor warming-responsive genes might not be influenced by LT treatment (Table S5).

#### 2.1.4. Genes Upregulated by Both Warming and Minor-Warming Treatment

Among DEGs, we attempted to identify genes that were upregulated by warming, minor warming, and both treatments (Table 1; Tables S6–S14). The numbers of genes upregulated under each condition were the same as those listed in Figure 1, Figure 2 and Figure 3, but 759 and 726 genes were upregulated by both conditions in Chiifu and Kenshin, respectively (Table S6). We reasoned that these genes might be involved in acquired thermotolerance and/or long-term adaptation to HT in both Chiifu and Kenshin. To specifically identify genes that might be involved in long-term adaptation to HT in the subtropical species Kenshin, we extracted specifically expressed genes (SEGs) from our data set (Tables S8–S13). We identified 85 genes that were specifically upregulated over 2-fold by both minor-warming and warming conditions in Chiifu but not in Kenshin (Table S10). In addition, 86 genes were specifically upregulated over 2-fold by both minor-warming and warming conditions in Kenshin but not in Chiifu (Table S13). Among these, 27 genes were nonannotated and unknown genes. Genes commonly upregulated in both Chiifu and Kenshin under both minor-warming and warming conditions included 15 *HSP*s and chaperone genes (Table S14). We subjected the genes listed in Tables S12–S14 to further GO enrichment analysis (Table 2, Table 3 and Table 4).

#### 2.1.5. Genes Upregulated by Both LT and Warming Treatment

Since both LT (transfer from 22 to 12 °C) and warming (transfer from 12 to 28 °C) are considered to be temperature-stress treatments, we analyzed genes responsive to LT and warming conditions (Table 1; Tables S7 and S15). We expected that genes upregulated by LT would also be upregulated by warming, but only a small number of genes in these categories overlapped: 40 and 59 genes in Chiifu and Kenshin, respectively (Table 1; Table S15). Interestingly, only one gene, *BrHSFA2* (Bra000557, an ortholog of AT2G26150), was upregulated under both conditions as well as in both inbred lines. Therefore, BrHSFA2 represents a candidate temperature-specific regulator in Chinese cabbage. 

In contrast to the single upregulated gene, many genes were downregulated by both LT and warming (Table S16). Whereas no gene was upregulated under both LT and warming conditions in Chiifu, 28 genes were downregulated under both conditions in Kenshin, implying that Kenshin is more sensitive to changes in temperature. The expression levels of these 28 genes were highest under normal growth conditions and lowest at 28 °C. Well-known genes in this category include genes encoding HY5-homolog (*HYH*; AT3G17609; Bra022225, Bra021258), gibberellin 2-oxidase 1 (*GA2OX1*; AT1G78440; Bra008362), CONSTANS-like 1 (*COL1*; AT5G15850; Bra023541), and CONSTANS-like 2 (*COL2*; AT3G02380; Bra021464, Bra001043).

### 2.2. GO Analysis of Warming- and Minor Warming-Responsive Genes

To identify genes associated with acclimation or adaptation to HT, we functionally classified upregulated genes in Kenshin under warming conditions, in Kenshin under both warming and minor-warming conditions, and in both lines under warming and minor-warming conditions (Tables S12–S14) via GO analysis (Table 2). Unexpectedly, no gene was identified in the “heat acclimation” or “response to heat” category among genes only expressed in Kenshin in response to warming conditions, but these categories were enriched among upregulated genes in both lines. Only one “heat acclimation”-related gene (*BrCYP71B2*) was identified in Kenshin under both minor-warming and warming conditions. However, several genes in putative HT adaptation-related gene categories were identified, such as 6 “heat acclimation”, 13 “response to heat”, and “chromosome organization” genes, among genes upregulated by warming and minor warming in both lines. Genes in the “heat acclimation” category included *BrHSP18.2*, *BrROF2* (FKBP-type peptidyl-prolyl *cis-trans* isomerase family protein), *BrHSP20-L*, *BrHSFA2*, *BrMge1* (*Mitochondrial GrpE2*), and an unknown gene. Genes in the “response to heat” category included most sHSPs, and “chromosome organization” genes included *BrHON4*, *BrSMP1* (*Swellmap 1*), *BrSWIB*, *BrENTG/VHS* family protein, and *BrSWC6* (SWR1 complex subunit 6) (Table S14). Categories that were specifically enriched in Kenshin under warming conditions included “lipid biosynthetic process”, “response to hormone stimulus” (most were auxin responsive), “intracellular membrane bound organelle” (most of unknown function), and “signal transduction” (most were defense related) (Table S12).

### 2.3. Identification of Genes Associated with HT Adaptation in Kenshin

To identify HT adaptation-related genes, we adopted several selection criteria based on the results shown in Table 2. The selection criteria were: (1) expression levels in Kenshin at 22 °C over 2-fold higher than those in Chiifu (we hypothesized that genes essential for long-term adaptation to HT would exhibit high basal levels of expression); and (2) expression levels under both warming and minor-warming conditions at least 2-fold higher in Kenshin than in Chiifu. Sixty-four genes were identified (Table 3), including 16 genes that were upregulated in both lines under minor-warming and warming conditions (asterisks in Table 3): 10 HSR genes, 2 TF (transcription factor) genes, 1 SF (splicing factor) gene, and 3 other genes. The two transcription factor genes were *BrPIF6* (Bra007660; phytochrome-interacting factor 3-like 2; PIL2/PIF6) and *Bra006853* (MYB-like transcription factor family protein). Genes in other categories included *Bra037453* (disease resistance protein (CC-NBS-LRR class) family) for “response to stress”, *BrGLN1.3* (Bra021276; glutamine synthetase 1.3) for “ligase activity”, and *BrROT3* (Bra011678; cytochrome P450 superfamily protein (ROT3)) for “lipid biosynthesis process”. These 16 genes might play important roles in HT adaptation in *B. rapa*. We subjected three of these genes to further analysis: *BrHSFA2*, *BrHSP18.2s*, and *BrSMP1* (Bra023741).

### 2.4. Comparison of HT-Related Gene Expression between B. rapa and Arabidopsis

Genes involved in thermotolerance (basal thermotolerance, acquired thermotolerance, and warming tolerance) and the associated marker genes are well known in the model plant *Arabidopsis*. To determine whether the same set of genes functions in *B. rapa*, we compared the expression patterns of these genes and other *HSP* genes with our microarray data (Table 4). Warming genes (*PHYB*, *HSP70*, and *PIF4*) identified in *Arabidopsis* were highly expressed in all *B. rapa* samples, with no notable increase in expression upon warming treatment, implying that genes responsible for long-term HT adaptation in *B. rapa* are different from *Arabidopsis* warming genes. In other cases, we assumed differences among samples, such as two contrasting lines in *B. rapa* vs. an ecotype of *Arabidopsis*. *BrPIF4* (Bra000283) expression appeared to be somewhat related to HT adaptation in *B. rapa*. Two Chinese cabbage genes homologous to acquired thermotolerance-related genes in *Arabidopsis*, *BrROF2* and *BrHSFA2*, appear to be critical for warming adaptation in *B. rapa*. The expression levels of several *HSP* genes were also consistent with warming treatment, pointing to their possible involvement in adaptation to HT.

### 2.5. Confirmation of Microarray Data via qRT-PCR

**Figure 5:**
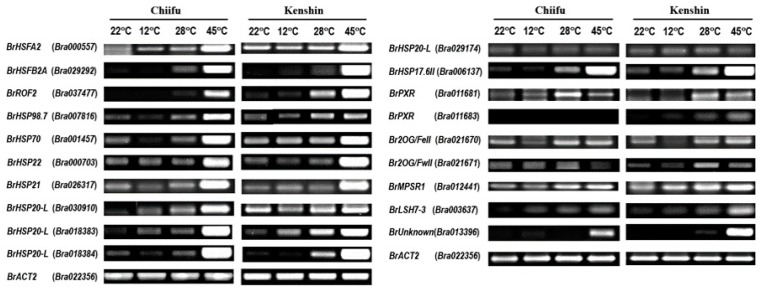
RT-PCR analysis of selected genes identified by microarray analysis. The expression levels of these genes obtained by microarray analysis are summarized in Table S16.

To confirm the expression levels of the genes detected by microarray analysis, we performed RT-PCR analysis of several selected genes (Figure 5). Although RT-PCR appears to be less sensitive than microarray analysis, RT-PCR results are often used to support microarray data. The expression levels of most of these genes increased upon warming treatment (28 °C), with maximum levels detected at 45 °C. These genes included three heat-acclimation-related genes (*BrHSFA2*, *BrHSFB2A*, and *BrROF2*), various *HSP* genes (especially *sHSP*s), peroxidase family genes, and others. Several genes showed high basal expression levels that further increased upon warming conditions: *BrHSP98.7*, *BrHSP70*, *BrHSP21*, three *BrHSP20L*s (Bra30910, Bra01883, Bra01884), and *BrMPSR1*. As expected, all of these genes showed higher basal expression levels in Kenshin than in Chiifu. 

### 2.6. Expression of BrHSFA2 and BrHSP18.2

Based on our data (Table 3 and Table 4) and previous reports [22,23,24], we selected *BrHSFA2* and *BrHSP18.2A-C* for further analysis of their possible involvement in response to warming and HT conditions. To examine whether *BrHSFA2* undergoes alternative splicing upon HT exposure, as does *Arabidopsis HSFA2*, and whether the intron sequences of this gene are the same in Kenshin and Chiifu, we cloned and sequenced at least 10 clones of the *BrHSFA2* intron region including part of Exon 1 and Exon 2 from both lines. These 548 bp fragments, including the 337 bp intron sequence, were 100% identical between Chiifu and Kenshin (NCBI accession MH310901, MH310902). We then compared *BrHSFA2* with the homologous sequence from *Arabidopsis* to investigate whether *BrHSFA2* also undergoes alternative splicing (Figure S1). As shown in Figure S1B, *BrHSFA2* might contain a mini-exon with a TAG stop codon, which produces a truncated version of the BrHSFA2 polypeptide via alternative splicing. The truncated version of BrHSFA2 has different C-terminal amino acids from *Arabidopsis* HSFA2 (Figure S1C). 

**Figure 6:**
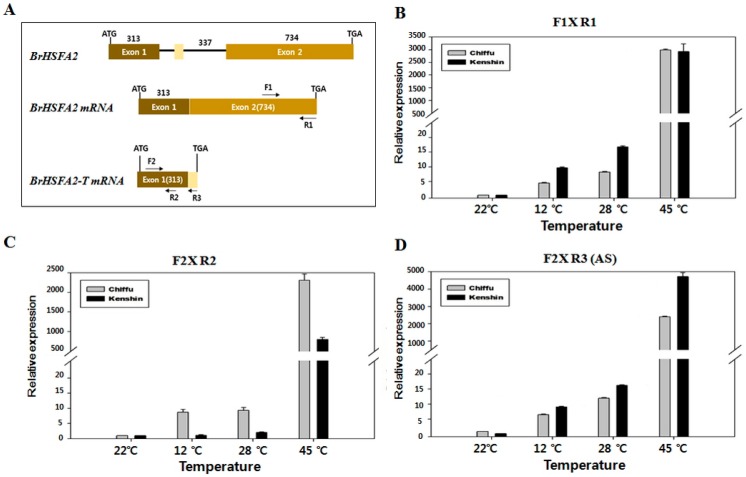
*BrHSFA2* expression in Chiifu and Kenshin during warming and heat shock treatments. qRT-PCR was performed with primer sets described in Table S1 and data analysis was carried out using qPCR value of three replicates. (**A**) Genomic organization of *BrHSFA2* and possible mRNAs with primer positions indicated; (**B**) Full-length *BrHSFA2* mRNA levels; (**C**) *BrHSFA2* mRNA containing both full-length and truncated (alternatively spliced) forms; (**D**) Truncated form of *BrHSFA2* mRNA.

To confirm that alternative splicing occurs in *BrHSFA2*, we carried out qRT-PCR with a reverse primer consisting of possible mini-exon-derived mRNA (Table S1; Figure 6). The levels of an alternatively spliced form of the transcript were higher in Kenshin than in Chiifu upon warming conditions (Figure 6D), while the total transcript levels (full-length + alternatively spliced form) were higher in Chiifu (Figure 6C). The level of full-length mRNA was higher in Kenshin than in Chiifu (Figure 6B). These results indicate that alternative splicing occurs in the intron of *BrHSFA2*, that this process facilitates the expression of full-length *BrHSFA2* as in *Arabidopsis*, and that the levels of the alternatively spliced form of this gene are higher in Kenshin (adapted to HT) than in Chiifu. To examine any association of the alternative splicing of *BrHSFA2* with *BrSMP1*, encoding a spliceosome component and a candidate gene involved in HT adaptation in Kenshin (Table 3), we examined the expression of *BrSMP1* under the same conditions (Figure S3). The expression pattern of *BrSMP1* upon warming and HS was proportional to *BrHSFA2* expression, suggesting the possible involvement of BrSMP1 in alternative splicing of *BrHSFA2* upon HT treatment.

HSFA2 is responsible for maintaining HS memory up to two days in *Arabidopsis* by maintaining histone methylation, thereby enabling the quick induction of HSR genes upon recurring HS [23,24]. HSFA2 has the most pronounced effect on *Arabidopsis HSP18.2* (*Hsp18.1-CI*/AT5G59720) [23]. *B. rapa* possesses three homologs corresponding to *AtHSP18.2*, *BrHSP18.2A* (Bra002539), *BrHSP18.2B* (Bra020295), and *BrHSP18.2C* (Bra006697), as listed in order from the highest to lowest identity with *AtHSP18.2*. These three genes encode highly identical polypeptides (93–96% identity) (Figure S2), but we successfully generated primer sets to distinguish each gene (Table S1). A gradual increase in temperature (by 5 °C every 2 h [HS]) strongly upregulated all three genes at 37 °C, with the greatest increase observed for *BrHSP18.2A* and *BrHSP18.2B* in Kenshin (Table 5). However, under warming conditions, compared with 27 °C during HS treatment, tremendous increases were observed in the expression of *BrHSP18.2A* and *BrHSP18.2B* in Kenshin, but there was a several-fold higher increase in *BrHSP18.2C* expression in Chiifu than in Kenshin. These results appear to reflect the different responses of *B. rapa* to HT acclimation compared with *Arabidopsis*, as well as differences between Kenshin and Chiifu.

### 2.7. BrHSP18.2 Promoter Analysis

The differential expression levels of the three *BrHSP18.2*s upon warming treatment prompted us to analyze cis-elements in their promoters. We designed a common forward primer based on a comparison of known *B. oleracea* genes, *B. napus* genes, three *B. rapa* genes (*BrHSP18.2A*: *Bra002539*, *BrHSP18.2B*: *Bra020295*, *BrHSP18.2C*: *Bra006697*), and AT5G59720, as well as specific reverse primers for each gene (Table S1). We obtained promoter sequences of different sizes (from the ATG start codon): 1257 bp for *BrHSP18.2A*, 969 bp for *BrHSP18.2B*, and 1199 and 1236 bp for *BrHSP18.2C*_Chiifu and Kenshin, respectively (NCBI accession MH310903-8). The promoter sequences of *BrHSP18.2A* and *B* were identical between Chiifu and Kenshin, but the promoter sequences of *BrHSP18.2C* were not similar between the two inbred lines. However, the promoters of the three genes were different from each other but with partially conserved regions.

Despite sequence difference among the *BrHSP18.2A*, *B*, and *C* promoters, four HSE-binding modules [25] were present between −53 and −194 upstream of the ATG start codon: two head-to-head (nGAAnnTTCn) and two tail-to-tail (nTTCnnGAAn) modules (Figure S4). Two modules at −194 to −180 were overlapping. In *Arabidopsis*, eight HSEs (a(g,t,c)GAAn, a(g,t,c)GnAn, or a(g,t,c)Gann) have been detected between −97 and −53 bp in *HSP18.2*, and six HSE deletions were detected, leading to a loss of promoter activity [26]. In *BrHSP18.2*s, seven HSEs were present. The finding that *BrHSP18.2A*, *B*, and *C* possess sufficient numbers of HSEs for HSF binding, as well as possessing identical HSEs, points to the importance of having sufficient numbers of HSFs (or other elements) to control *HSP* expression levels.

## 3. Discussion

Plants exposed to HT exhibit reduced growth and development, as well as changes in signaling cascades or gene expression, representing adaptive responses to HT [27]. Global climate change has prompted breeders to develop thermotolerant crop varieties, including vegetable crops. Identifying genes that regulate plant responses to warming (or HT adaptation) and elucidating the mechanisms underlying their functions will be crucial for coping with the effects of global warming on agriculture. To identify and characterize warming-associated genes from Chinese cabbage, we subjected Chiifu and Kenshin, two contrasting inbred lines with respect to geographic origin and temperature responsiveness, to transcriptome analysis with a newly developed 3′-tiling microarray (135 K) covering the whole *Brassica rapa* genome using newly designed temperature treatments. Although the warming condition is different from the heat stress, some of HSR genes like *sHSP*s showed similar upregulated patterns as described in previous work [17]. However, this analysis yielded several novel findings, including the discovery of putative HT-adaptive genes, alternative splicing of *BrHSFA2*, and the expression patterns of its target genes.

### 3.1. Transcriptome Analysis

Of the three treatments, both warming and minor-warming conditions upregulated genes enriched in five biological process categories in both inbred lines, including “response to heat”, “heat acclimation”, and “response to temperature stimulus” (Figure 2 and Figure 3), indicating that the general responses of both lines are similar. However, genes involved in “response to auxin stimulus” were upregulated by both warming and minor warming in Kenshin, but not in Chiifu. This category of genes might be important for HT adaptation in Chinese cabbage. There is increasing evidence for an association between HT and auxin responses—HT reduces auxin biosynthesis [28], and HSPs such as sHSP22 [29] and cytosolic HSP90 [30] regulate auxin responses.

Our transcriptome analysis indicated that *BrHSFA2* was upregulated or induced in both lines under all three treatment conditions (LT, warming, and minor warming). *Arabidopsis HSFA2* is induced by HT and plays a role in the maintenance of HS memory [26,31,32]. *Arabidopsis HSFA2* expression is also associated with H_2_O_2_/ROS stress [33] and salt/osmotic stress [34], suggesting that it plays an important role in responses to various stress conditions. The induction of *BrHSFA2* expression by LT treatment suggests that BrHSFA2 targets LT-responsive genes besides HSR genes upon exposure to HT. 

### 3.2. Functional Classification of Putative Warming Genes

As shown in Table 2 and Tables S12–S14, most genes belonging to the GO categories “heat acclimation”, “response to heat”, and “chromosome organization” were upregulated in both inbred lines, but their levels were higher in Kenshin than in Chiifu. These results suggest that the minor differences in expression of these genes in *B. rapa* could result from long-term adaptation to HS or that the genes identified in *Arabidopsis* could be associated with short-term HS adaptation. *BrCYP71B2* (involved in “heat acclimation”) was upregulated in Kenshin under both minor-warming and warming conditions; its homolog is also upregulated upon HS in *Arabidopsis* [35], indicating its possible involvement in the HSR in *B. rapa*. Some putative HT adaptation-related genes have well-known functions in *Arabidopsis*. For example, *Mge1* is induced by heat (under the control of HsfA1) and confers thermotolerance under priming conditions [36]. *Arabidopsis* SMP1 and SWC6 are associated with splicing [37] and chromatin remodeling [38], respectively. These findings suggest that these genes might play a role in HT adaptation in Kenshin. Kenshin-specific genes were classified into four categories (“lipid biosynthetic process”, “response to hormone stimulus”, “intracellular membrane bound organelle”, and “signal transduction”) (Table 2; Table S12), but none were found to be involved in HT adaptation, except for auxin-responsive genes. Further studies are needed to investigate the roles of these genes in *B. rapa*.

### 3.3. Putative HT Adaptation-Related Genes in Kenshin

To identify and characterize putative HT adaptation-related genes in Kenshin, we applied new cutoff criteria (Table 3) compared with known genes from *Arabidopsis* (Table 4) and confirmed their expression by RT-PCR (Figure 5). Unexpectedly, *B. rapa* warming genes appeared to differ from those of *Arabidopsis*, suggesting that different warming adaptation mechanisms or different sets of genes might function in *B. rapa* upon warming conditions. *Arabidopsis* warming genes such as *PHYB*, *HSP70*, and *PIF4* were highly expressed in all *B. rapa* samples, with no notable increase upon warming treatment. Only the expression pattern of *BrPIF4* (Bra000283) appeared to be somewhat related to HT adaptation, and two homologs of acquired thermotolerance-related genes in *Arabidopsis*, *BrROF2* and *BrHSFA2*, appeared to be responsive to warming in *B. rapa*. PIF4, a basic helix-loop-helix (bHLH) transcription factor, is a central regulator of ambient temperature signaling in *Arabidopsis* [9]. PIF4-mediated thermomorphogenesis is associated with the circadian clock [39,40], auxin [9,41,42], other phytohormones [43,44], and epigenetic modification [6]. Quint et al. (2016) [11] indicated that PIF controls thermomorphogenesis via three molecular circuitries: (1) transcriptional regulation of circadian clock genes; (2) post-translation regulation by phosphorylation and degradation; and (3) phytohormonal control through interactions at various levels. These findings and our expression data suggest that BrPIF4 plays a role in the adaptation of Kenshin to HT.

Most warming-responsive genes in Kenshin are orthologs of *Arabidopsis* genes involved in acquired thermotolerance: HSR genes, s*HSP*s, peroxidase family genes, and disease-resistance genes (Table 3; Figure 5). The expression of other *HSP* genes and *BrMPSR1* also increased in response to warming conditions, suggesting their possible involvement in HT adaptation (Figure 5). The roles of a few s*HSP*s in heat tolerance have been examined, including genes encoding HSP 21 (*HSP21*; Bra026317, AT4G27670) [45] and 17.6 kDa class II HSP (*HSP17.6II*; AT5G12020; Bra006137, Bra008920) [29]. However, many s*HSP*s, such as *HSP21*, *HSP22.0*, *HSP18.2*, and *ASCORBATE PEROXIDASE 2* (*APX2*), are HS memory-related genes [22] and are targeted by HSFA2 to help maintain HS memory [31,32]. Class III peroxidases (PRXs) are plant-specific enzymes encoded by multigene families that are involved in lignification, cell elongation, stress responses, and seed germination [46]. Ascorbate peroxidase (APX), a key antioxidant enzyme, participates in various abiotic stress responses and in maintaining cellular homeostasis [47]. In *Arabidopsis*, MPSR1 (Misfolded Protein Sensing RING E3 Ligase 1) is involved in the rapid degradation of misfolded proteins due to protein-damaging stress, thereby controlling proteotoxic stress in the cytoplasm [48]. In *B. rapa*, two *MPSR1* genes (*BrMPSR1-1* (*Bra012441*) and *BrMPSR1-2*(*Bra016290*)) appear to be regulated at the transcriptional level or regulated in evolutionarily divergent ways. These genes might also participate in HT tolerance in Kenshin. Together, these findings support the notion that these genes play a role in long-term adaptation to HT in Kenshin.

### 3.4. BrHSFA2 and Its Target BrHSP18.2s

The expression of *Arabidopsis HSFA2* is dependent on HS (the expression of which is amplified by the production of its alternatively spliced form), increases the expression of target HSR genes such as *HSP18.2*, and confers acquired thermotolerance or HS memory. In the current study, *BrHSFA2* expression and splicing, and the expression of its target gene, *BrHSP18.2s* (Figure 6; Table 5), followed a similar pattern to that of *Arabidopsis* under warming conditions, implying that the warming response of *B. rapa* is similar to acquired thermotolerance in *Arabidopsis*.

Acquired thermotolerance by exposure to moderate HS confers tolerance to normally lethal HT [7]; this thermotolerance is maintained as HS memory for several days [32,49,50]. Three HS memory maintenance-related genes have been identified in *Arabidopsis*, which maintain this memory for several days after the plant returns to nonstress temperature conditions: two days for *HSFA2* [23,24,28], three days for heat stress-associated 32 kD protein gene (*Hsa32*) [49], and three days for *miR156* [22]. The maintenance of HS memory results from the induced hypermethylation of target genes (*HSR* genes), although not all target genes are hypermethylated [51].

*Arabidopsis* HSFA2 is a key regulator of responses to various types of stress including heat, high light, and ROS stress and is required for extending acquired thermotolerance by maintaining the expression of *HSP* genes [26,28,31]. HSFA2 is a regulatory component responsive to the accumulation of misfolded proteins in the cytosol [52]. HSFA2 also induces abscisic-acid-mediated heat tolerance by upregulating *HSPs* in both a monocot (fescue) and a dicot (*Arabidopsis*) [53]. HSFA2 is responsible for maintaining HS memory up to two days by maintaining histone methylation, thereby inducing HSR gene expression upon recurring HS [23,24]. Many small *HSP* genes, such as *HSP21*, *HSP22.0*, *HSP18.2*, and *ASCORBATE PEROXIDASE 2* (*APX2*), are HS memory-related genes in *Arabidopsis* [22,24], whereas *Hsp70* (AT3G12580) and *Hsp101* (AT1G74310) are non-HS memory-related genes [24]. *Arabidopsis HSFA2* produces an alternatively spliced form (truncated form) upon HS, and this truncated form in turn increases *HSFA2* transcription levels [54]. This scenario appears to operate in *B. rapa* as well, where *BrHSFA2* undergoes alternative splicing, is upregulated, and induces/maintains target gene expression/memory. Alternative splicing by a spliceosome complex is an important mechanism in the sensing of (and adaptation to) small variations in ambient temperature [14], as well as acquired thermotolerance conditions [48], in *Arabidopsis*. These findings imply that temperature changes, including HS and priming, lead to alternative splicing. Alternative splicing might contribute to long-term adaptation to HT in Chinese cabbage, in which thermotolerance does not appear to be due to morphological and architectural changes caused by high ambient temperatures, as found in other plants (thermomorphogenesis) [11].

### 3.5. BrHSP18.2s Promoters and Their Possible Control

The induction of *Arabidopsis HSP18.2* (*Hsp18.1-CI*/AT5G59720) expression by HSFA2, a major thermotolerance HSF [24,26,31], is related to the role of HSFA2 in sustaining H3K4 methylation [24]. The promoter activity of *ArabidopsisHSP18.2* is highest at 35 °C [55]. This promoter contains eight HSE modules between −97 and −53 bp; the deletion of two modules maintains promoter activity, but a deletion of six modules causes a dramatic reduction in promoter activity [26]. All *BrHSP18.2* promoters contain seven HSE modules, implying that there is no difference in BrHSFA2 binding among the three *BrHSP18.2* promoters. In other organisms, at least two nGAAn units arranged head-to-head (nGAAnnTTCn) or tail-to-tail (nTTCnnGAAn) are required in the promoters of *HSPs* for efficient HSF binding [25]. Four HSE units were found in all *BrHSP18.2s* promoters, one of which is overlapping (Figure S4), indicating that *BrHSP18.2A* to *C* contain sufficient numbers of HSEs for TF binding. The binding of HSFA2 with several target genes including *HSP18.2* has been assessed [26], showing that two modules of a TATA-proximal HSE (nGAAnnTTCn) are essential for transcriptional activation by HSFA2. These modules are also conserved in all *BrHSP18.2s* promoters, suggesting that the expression differences among *BrHSP18.2s* upon HT exposure or warming conditions might be due to the presence of different numbers of HSFs such as *BrHSFA2* (which is controlled by alternative splicing) and/or other factor(s).

## 4. Materials and Methods

### 4.1. Plant Materials

Seeds of two Chinese cabbage (*Brassica rapa* ssp. *pekinensis*) inbred lines, Chiifu and Kenshin, were kindly provided by Woori Seed Co., Sejong City, Korea. The seeds were sown in a 32-hole tray (6 × 6 × 6 cm × 32 holes) and grown for approximately 3 weeks in a growth chamber at 22 °C under a 16 h light/8 h dark photoperiod with a photon flux density of 140 μmol m^−2^ s^−1^. For warming treatment, the plants were subjected to 12 °C for 2 days under the same photoperiod and transferred to 28 °C for 3 h. For extreme HS treatment, warming-exposed plants were further incubated at 45 °C in a growth chamber for 3 h. The samples were collected at the end of each treatment (Figure 1). Humidity of growth chambers was set to 70 ± 10%. Shoots from five individual plants were sampled and quickly frozen in liquid nitrogen. To prepare the other experimental samples, plants grown for 3 weeks were subjected to various temperature treatments.

### 4.2. Br135K Microarray Analysis

The Br135K microarray (Brapa_V3_microarray, 3′-Tiling microarray) is a high-density DNA array prepared with Maskless Array Synthesizer (MAS) technology by NimbleGen (http://www.nimblegen.com/) [55]. Probes were designed from 41,173 genes from *Brassica rapa* accession Chiifu-401-42 (http://brassicadb.org/brad/). All three probes were 60 mers with 30 bp overlaps in 120 bp regions (60 bp of coding sequence plus 60 bp of the 3’UTR for each gene), representing 123,647 features. Fifty features from five markers (*GUS*, *GFP*, *Bar*, *Kan*, *Hyg*) were also included. Total and polysomal RNA were extracted using an RNeasy Mini kit (Qiagen, GmbH, Hilden, Germany) and the RNA protect Reagent (Qiagen), and contaminating DNA was removed by on-column DNase digestion with RNase-free DNase (Promega, Madison, WI, USA). Labeling, data processing, and background correction were performed as described previously [56]. To assess the reproducibility of the microarray analysis, the experiment was repeated using independently prepared total RNA samples from two biological replicates. To obtain insights regarding the putative biological functions and biochemical pathways of the DEGs, enrichment analysis was carried out by searching the GO [57], agriGO [58], and Kyoto Encyclopedia of Genes and Genomes [59] databases.

### 4.3. RNA Extraction, RT-PCR, and qRT-PCR

Total RNA was extracted from the plant samples using an RNeasy Mini kit (Qiagen). The RNA was treated with RNase-free DNase (Promega) to remove genomic DNA contamination. RT-PCR was performed using an Avian Myeloblastosis Virus (AMV) One-step RT-PCR kit (Takara, Kusatsu, Shiga, Japan). The gene-specific primers used to analyze the selected genes are listed in Table S1. For qRT-PCR, the RNA was subjected to first-strand cDNA synthesis using an Ace-α kit with Oligo-dT primers (Toyobo, Osaka, Japan). The primer sequences were designed according to sequences from the *Brassica* database (BRAD, http://brassicadb.org/brad/). PCR was performed using SYBR^®^ Green Realtime PCR Master Mix-Plus (Toyobo, Japan) under the following cycling conditions: 30 s at 95 °C followed by 30 cycles of 95 °C for 5 s, 58 °C for 10 s, and 72 °C for 15 s.

### 4.4. Gene Cloning and Sequence Analysis

To analyze the intron sequence of *BrHSFA2* and promoter sequences of *B. rapa* small *HSP18.2* genes (*BrHSP18.2*s), genomic DNA was cloned and analyzed. Genomic DNA was isolated from Chiifu and Kenshin leaves using a DNeasy Plant Mini kit (Qiagen GmbH, Hilden, Germany). Primers were designed based on sequences listed in the BRAD website (Table S1). Genomic PCR was performed under the following conditions: denaturation (5 min at 94 °C), 30 cycles of amplification (30 s at 94 °C, 30 s at 52 °C, and 3 min at 72 °C), and a final extension (7 min at 72 °C). The PCR products were purified using a MEGA-Spin Gel Extraction kit (Intron Biotech. Inc., Sungnam, Korea) and cloned into the TA-vector using a T&A Cloning kit (RBC Bioscience Corp., New Taipei City, Taiwan). *Escherichia coli* (DH5α) cells were transformed with plasmid DNA carrying the desired insert. Plasmid DNA was purified using DNA-Spin (Intron Biotech. Inc., Sungnam, Korea) prior to sequencing (Macrogen, Seoul, Korea). To eliminate PCR and sequencing errors, at least 10 clones per gene were sequenced and analyzed. Any possible PCR and/or sequencing errors were eliminated by aligning independent sequences (http://www.genome.jp/tools-bin/clustalw).

## 5. Conclusions

This is the first report of the effects of long-term adaptation to warmer growth conditions or HT-adaptation of the gene expression profile in crops. We were able to derive the following conclusions from this study. Many DEGs were overlapping between minor-warming and warming conditions and in both lines examined, Chiifu and Kenshin. Most HT adaptation-associated genes in Chinese cabbage are homologous to acquired thermotolerance-related genes in *Arabidopsis*. Sixteen putative HT adaptation-related genes were identified: 10 HSR genes (including *BrHSFA2* and *sHSP*s), 2 TF genes (*BrPIF6* and a *BrMyB*), 1 SF gene (Pre-mRNA splicing Prp18-interacting factor, *BrSMP1*), and 3 other genes. Three additional genes, *BrPIF4*, *BrROF2*, and *BrMPSR1*, were also identified as candidate genes involved in HT adaptation. The degree of expression of *HSR* genes such as *BrHSP18.2*s appears to be related to the levels of HSF protein such as BrHSFA2 rather than their own promoter activity. Adaptation to HT in Chinese cabbage appears to be due to changes in the auxin response, increases in the alternative splicing of *BrHSFA2* to amplify its expression, HS memory of HSR genes, and their increases in expression upon recurring HT. The genes identified in this study could be utilized in molecular breeding and marker development after further analysis.

## Figures and Tables

**Table 1:** Summary of genes upregulated by various treatments. SEGs (specifically expressed genes) represent genes showing an over 2-fold change in expression under the indicated condition but no change or downregulation under the other conditions.

Inbred Line (Treatment)	Comparison	No. of Genes (Table S5)	SEGs (Tables S7–S12)	Comparison	No. of Genes (Table S6)
Chiifu (22 °C ⟶ 12 °C ⟶ 28 °C)	28 °C/22 °C	1651	121	12 °C/22 °C	1532
	28 °C/12 °C	2010	49	28 °C/12 °C	2010
	Both	759	85	Both	40
Kenshin (22 °C ⟶ 12 °C ⟶ 28 °C)	28 °C/22 °C	1841	193	12 °C/22 °C	1669
	28 °C/12 °C	2093	146	28 °C/12 °C	2093
	Both	726	86	Both	59

12 °C/22 °C: LT conditions; 28 °C/12 °C: warming conditions; 28 °C/22 °C: minor-warming conditions.

**Table 2:** Functional classification of genes specifically expressed in response to HT acclimation and/or adaption conditions. The table was constructed based on Tables S11–S13 using agriGO (http://bioinfo.cau.edu.cn/agriGO/) based on GO information for *Arabidopsis* homologs. W, warming, MW, minor warming.

Classification	Kenshin (W)	Kenshin (W + MW)	Kenshin/Chiifu (W + MW)
Heat acclimation	-	1	6
Response to heat	-	-	13
Response to stress	8	6	8
Transcription factor activity	9	9	9
Transferase activity	9	5	12
Transport	7	6	6
Carbohydrate metabolic process	8	4	4
Ligase activity	-	5	1
Lipid biosynthetic process	14	4	3
Oxidation reduction	-	1	2
Response to auxin stimulus	-	3	1
Response to oxidative stress	-	3	2
Response to salicylic acid stimulus	-	3	3
Chromosome organization	-	-	5
Response to hormone stimulus	22	-	-
Intracellular membrane bound organelle	23	-	-
Signal transduction	6	-	-
Primary metabolic process	7	-	-
Catalytic activity	7	-	-
Plasma membrane	3	-	-
Ion binding	5	-	-
Unclassified	4	15	34
Unknown protein	6	5	14
Not annotated	8	16	34
Total	132	86	157

**Table 3:** Genes associated with the adaptation of Kenshin to HT based on microarray analysis. The selection criteria were (1) intrinsic levels of expression in Kenshin at 22 °C over 2-fold higher than those in Chiifu; and (2) expression levels under both warming and minor-warming conditions at least 2-fold higher in Kenshin than in Chiifu. HSR, heat shock response; TF, transcription factor; SF, splicing factor.

Classification	*At*_Locus	Gene Description	*Br*_SEQ_ID	Expression Level (Probe Intensity)						Fold Change			
				Chiifu (C)			Kenshin (K)			Intrinsic	Warming	Warming	Minor-W
				22 °C	12 °C	28 °C	22 °C	12 °C	28 °C	K22/C22	K28/K12	C28/C12	K28/K22
**HSR**	AT2G26150	Heat shock transcription factor A2 (HSFA2)	*Bra000557* *	68	223	1256	256	542	1786	3.8	3.3	5.6	7.0
	AT5G62020	Heat shock transcription factor B2A (HSFB2A)	*Bra029292* *	385	374	3164	875	587	3814	2.3	6.5	8.5	4.4
	AT4G25200	Mitochondrion-localized small heat shock protein 23.6 (HSP23.6-MITO)	*Bra013872* *	333	323	1308	984	265	3551	3.0	13.4	4.0	3.6
	AT4G10250	HSP20-like chaperones superfamily protein (HSP22.0)	*Bra027999* *	27	71	378	85	37	677	3.2	18.1	5.3	7.9
	AT1G54050	HSP20-like chaperones superfamily protein	*Bra030910* *	827	945	4041	2406	2649	9204	2.9	3.5	4.3	3.8
	AT2G29500	HSP20-like chaperones superfamily protein	*Bra018383* *	1870	2774	11,166	5211	2428	19,026	2.8	7.8	4.0	3.7
			*Bra018384*	2575	5095	6789	5906	5362	14,692	2.3	2.7	1.3	2.5
			*Bra031725*	1996	4409	5909	6292	5275	13,450	3.2	2.5	1.3	2.1
	AT5G51440	HSP20-like chaperones superfamily protein	*Bra029174* *	406	376	5772	1657	292	7627	4.1	26.1	15.3	4.6
	AT5G59720	Heat shock protein 18.2 (HSP18.2)	*Bra002539* *	73	259	747	206	58	5114	2.8	88.5	2.9	24.8
	AT5G12020	17.6 kDa class II heat shock protein (HSP17.6II)	*Bra006137* *	52	271	2513	909	310	2036	17.6	6.6	9.3	2.2
	AT5G48570	FKBP-type peptidyl-prolyl *cis*-*trans* isomerase family protein (ROF2)	*Bra037477* *	309	283	4967	1632	352	6607	5.3	18.8	17.6	4.0
	AT1G72660	P-loop containing nucleoside triphosphate hydrolases superfamily protein	*Bra016043* *	212	185	2034	967	889	2723	4.6	3.1	11.0	2.8
	AT5G47830	Unknown protein	*Bra020728* *	523	544	4910	1310	1076	4854	2.5	4.5	9.0	3.7
	AT3G14200	Chaperone DnaJ-domain superfamily protein	*Bra027363* *	1333	2129	5365	2857	2634	7477	2.1	2.8	2.5	2.6
**TF**	AT2G23690	HTH-type transcriptional regulator	*Bra039208*	1122	1383	3449	2567	1072	6076	2.3	5.7	2.5	2.4
	AT5G56840	MYB-like transcription factor family protein	*Bra002790* *	89	154	589	251	144	767	2.8	5.3	3.8	3.0
	AT5G52600	MYB domain protein 82 (MYB82)	*Bra029113*	64	334	132	205	371	1579	3.2	4.3	0.4	7.7
	AT2G24645	Transcriptional factor B3 family protein	*Bra032079*	43	85	77	182	244	1013	4.3	4.2	0.9	5.6
	AT1G70270	Transcription factor	*Bra007905*	246	797	368	1127	952	3529	4.6	3.7	0.5	3.1
	AT5G15150	Homeobox 3 (HB3)	*Bra023506*	197	389	165	389	372	1184	2.0	3.2	0.4	3.0
	AT5G66940	Dof-type zinc finger DNA-binding family protein	*Bra012119*	488	1459	209	1159	983	2746	2.4	2.8	0.1	2.4
	AT3G62090	Phytochrome interacting factor 3-like 2 (PIL2/PIF6)	*Bra007660* *	218	239	537	426	684	1883	2.0	2.8	2.2	4.4
	AT5G10970	C2H2 and C2HC zinc fingers superfamily protein	*Bra009000*	303	776	668	823	878	2180	2.7	2.5	0.9	2.6
	AT1G23380	KNOTTED1-like homeobox gene 6 (KNAT6)	*Bra016348*	140	970	475	273	1004	2202	2.0	2.2	0.5	8.1
	AT4G18610	Light-dependent short hypocotyl 9 (LSH9)	*Bra021000*	83	85	77	189	112	605	2.3	5.4	0.9	3.2
	AT2G42610	Light-dependent short hypocotyl 10 (LSH10)	*Bra016865*	422	617	197	1008	1013	2079	2.4	2.1	0.3	2.1
**SF**	AT1G65660	Pre-mRNA splicing Prp18-interacting factor (SMP1)	*Bra023741*	141	232	1190	664	589	1712	4.7	2.9	5.1	2.6
**Others**	AT4G36430	Peroxidase superfamily protein	*Bra017761*	125	381	182	543	686	2278	4.4	3.3	0.5	4.2
	AT1G16530	ASYMMETRIC LEAVES 2-like 9 (LBD3/ASL9)	*Bra026042*	171	393	272	522	824	1729	3.1	2.1	0.7	3.3
			*Bra026716*	209	325	291	454	534	1063	2.2	2.0	0.9	2.3
	AT5G59670	Leucine-rich repeat protein kinase family protein	*Bra020300*	129	93	80	288	147	622	2.2	4.2	0.9	2.2
	AT4G19530	Disease resistance protein (TIR-NBS-LRR class) family	*Bra027594*	158	206	202	532	536	1591	3.4	3.0	1.0	3.0
	AT2G32660	Receptor like protein 22 (RLP22)	*Bra021803*	163	72	201	960	963	2013	5.9	2.1	2.8	2.1
	AT1G51860	Leucine-rich repeat protein kinase family protein	*Bra030411*	92	75	41	874	1051	1992	9.5	1.9	0.5	2.3
	AT1G53350	Disease resistance protein (CC-NBS-LRR class) family	*Bra037453* *	185	155	420	539	706	1425	2.9	2.0	2.7	2.6
	AT4G08570	Heavy metal transport/detoxification superfamily protein	*Bra037865*	37	45	505	255	67	2044	6.9	30.5	11.1	8.0
	AT5G66110	Heavy metal transport/detoxification superfamily protein (HIPP27)	*Bra009662*	115	329	449	1102	1195	7086	9.5	5.9	1.4	6.4
	AT1G79360	Organic cation/carnitine transporter 2 (OCT2)	*Bra035111*	53	37	23	135	111	568	2.5	5.1	0.6	4.2
	AT2G04100	MATE efflux family protein	*Bra015133*	78	181	255	350	510	1175	4.5	2.3	1.4	3.4
	AT2G35460	Late embryogenesis abundant (LEA) hydroxyproline-rich glycoprotein family	*Bra028562*	215	78	879	575	123	2595	2.7	21.1	11.3	4.5
	AT2G25450	2-oxoglutarate (2OG) and Fe(II)-dependent oxygenase superfamily protein	*Bra021671*	246	68	240	530	644	5146	2.2	8.0	3.5	9.7
	AT1G28030	2-oxoglutarate (2OG) and Fe(II)-dependent oxygenase superfamily protein	*Bra021552*	59	73	121	129	169	1079	2.2	6.4	1.7	8.4
	AT2G39310	Jacalin-related lectin 22 (JAL22)	*Bra005053*	16	99	45	36	109	826	2.3	7.6	0.5	23.1
	AT3G16900	LURP-one-like protein	*Bra021211*	217	205	358	1035	524	2674	4.8	5.1	1.7	2.6
	AT4G36380	Cytochrome P450 superfamily protein (ROT3)	*Bra011678* *	52	139	350	395	205	842	7.6	4.1	2.5	2.1
	AT4G24110	NADP-specific glutamate dehydrogenase	*Bra013763*	92	79	129	204	176	637	2.2	3.6	1.6	3.1
	AT3G51000	Alpha/beta-Hydrolases superfamily protein	*Bra036841*	69	21	23	676	873	3074	9.8	3.5	1.1	4.5
	AT4G22460	Bifunctional inhibitor/lipid-transfer protein/seed storage 2S albumin superfamily protein	*Bra013619*	152	251	304	541	555	1932	3.6	3.5	1.2	3.6
	AT4G13410	Nucleotide-diphospho-sugar transferases superfamily protein (CSLA15)	*Bra008638*	276	385	600	656	523	1384	2.4	2.6	1.6	2.1
	AT5G40650	Succinate dehydrogenase 2-2 (SDH2-2)	*Bra028469*	468	531	545	2517	2395	6064	5.4	2.5	1.0	2.4
	AT5G05390	Laccase 12 (LAC12)	*Bra009111*	279	1072	1201	1118	1176	2906	4.0	2.5	1.1	2.6
	AT3G17820	Glutamine synthetase 1.3 (GLN1.3)	*Bra021276* *	1197	1053	2579	2798	3121	7326	2.3	2.3	2.4	2.6
	AT5G61260	Plant calmodulin-binding protein-related	*Bra029324*	256	194	163	811	1078	2480	3.2	2.3	0.8	3.1
	AT5G64870	SPFH/Band 7/PHB domain-containing membrane-associated protein family	*Bra024333*	270	569	701	559	484	1113	2.1	2.3	1.2	2.0
	AT1G20575	Nucleotide-diphospho-sugar transferases superfamily protein	*Bra025828*	1293	888	2710	3467	3248	7310	2.7	2.3	3.1	2.1
	AT4G39140	RING/U-box superfamily protein	*Bra025860*	27	27	59	2422	2404	5394	88.5	2.2	2.2	2.2
	AT3G09260	Glycosyl hydrolase superfamily protein (BGLU23)	*Bra034060*	278	412	393	1034	995	2106	3.7	2.1	1.0	2.0
	AT3G06550	O-acetyltransferase family protein	*Bra040276*	250	250	310	492	762	1570	2.0	2.1	1.2	3.2
	AT1G29590	Eukaryotic initiation factor 4E protein (eIF4E3)	*Bra032325*	299	445	486	1304	1365	2571	4.4	1.9	1.1	2.0
	AT4G19430	Unknown protein	*Bra013396*	103	16	42	252	17	726	2.5	41.6	2.7	2.9
	NA	NA	*Bra012220*	183	112	97	5190	4062	11,440	28.4	2.8	0.9	2.2
	NA	NA	*Bra025861*	53	59	147	1979	1928	4522	37.3	2.3	2.5	2.3
	NA	NA	*Bra010352*	596	604	1785	4135	4187	9370	6.9	2.2	3.0	2.3

* Gene in Table S13 (genes upregulated in both Chiifu and Kenshin under both minor-warming and warming conditions).

**Table 4:** Summary of the expression levels of *B. rapa* genes shown to be HT responsive in *Arabidopsis*.

Marker	At_Locus	Gene Description	*Br*_SEQ_ID	Expression Level (Probe Intensity)						Fold Change			
				Chiifu			Kenshin			Chiifu		Kenshin	
				22 °C	12 °C	28 °C	22 °C	12 °C	28 °C	28/22 °C	28/12 °C	28/22 °C	28/12 °C
**Basal thermotolerance**	AT1G74310	Heat shock protein 101 (HSP101)	*Bra003807*	1457	1411	3457	1696	2238	3594	2.4	2.5	2.1	1.6
			*Bra015922*	2725	3674	6053	5409	5601	8821	2.2	1.6	1.6	1.6
	AT5G17020	Exportin 1A (XPO1A)	*Bra006382*	9010	8711	9368	8345	9747	11,271	1.0	1.1	1.4	1.2
			*Bra008580*	10,093	10,241	10,261	9834	8047	12,608	1.0	1.0	1.3	1.6
			*Bra023593*	6942	7056	7948	6939	6511	7561	1.1	1.1	1.1	1.2
**Acquired thermotolerance**	AT3G25230	Rotamase FKBP 1 (ROF1/FKBP62)	*Bra013224*	11	18	42	15	59	40	3.8	2.4	2.7	0.7
	AT5G48570	Rotamase FKBP 2 (ROF2/FKBP65)	*Bra037477* *	309	283	4967	1632	352	6607	16.1	17.6	4.0	18.8
	AT2G26150	Heat shock transcription factor A2 (HSFA2)	*Bra000557* *	68	223	1256	256	542	1786	18.5	5.6	7.0	3.3
**Warming**	AT2G18790	Phytochrome B (PHYB)	*Bra001650*	717	770	829	402	1083	527	1.2	1.1	1.3	0.5
			*Bra022192*	13,874	10,184	7989	16,575	11,087	13,685	0.6	0.8	0.8	1.2
	AT3G12580	Heat shock protein 70 (HSP70)	*Bra001457*	1560	2512	8099	9758	6732	13,032	5.2	3.2	1.3	1.9
			*Bra038734*	1049	1159	7871	6488	4093	7597	7.5	6.8	1.2	1.9
	AT2G43010	Phytochrome interacting factor 4 (PIF4)	*Bra000283* *	19,533	12,261	15,413	13,610	9359	24,186	0.8	1.3	1.8	2.6
			*Bra037742*	3718	4145	3350	1988	3218	4296	0.9	0.8	2.2	1.3
**Other HSPs**	AT2G25140	Casein lytic proteinase B4 (HSP98.7/CLPB4)	*Bra007816*	645	702	1353	1332	1269	2425	2.1	1.9	1.8	1.9
	AT4G16660	HSP 70 family protein	*Bra038496*	1160	1868	2286	1503	2091	1505	2.0	1.2	1.0	0.7
	AT4G25200	Mitochondrion-localized small heat shock protein 23.6 (HSP23.6-MITO)	*Bra013872* *	333	323	1308	984	265	3551	3.9	4.0	3.6	13.4
	AT4G10250	HSP20-like chaperones superfamily protein (HSP22.0-L)	*Bra000703* *	1174	1055	1638	1352	2898	6069	1.4	1.6	4.5	2.1
			*Bra027999* *	27	71	378	85	37	677	14.1	5.3	7.9	18.1
	AT4G27670	Heat shock protein 21 (HSP21)	*Bra026317*	45	166	729	310	54	603	16.1	4.4	1.9	11.2
	AT5G47590	Heat shock protein HSP20/alpha crystallin family	*Bra022051*	1119	1560	1914	1045	3295	2588	1.7	1.2	2.5	0.8
			*Bra022079*	4462	6564	7455	4309	9320	12,845	1.7	1.1	3.0	1.4
			*Bra022083*	1201	1103	1815	939	1902	1852	1.5	1.6	2.0	1.0
			*Bra022084*	1042	1120	1737	760	1635	1540	1.7	1.6	2.0	0.9
	AT1G53540	HSP20-like chaperones superfamily protein	*Bra018216* *	10,773	3454	10,773	7716	3159	16,904	1.0	3.1	2.2	5.4
	AT1G54050	HSP20-like chaperones superfamily protein	*Bra012949*	5473	4945	10796	1568	1799	1843	2.0	2.2	1.2	1.0
			*Bra030910* *	827	945	4041	2406	2649	9204	4.9	4.3	3.8	3.5
	AT2G29500	HSP20-like chaperones superfamily protein	*Bra018383* *	1870	2774	11,166	5211	2428	19,026	6.0	4.0	3.7	7.8
			*Bra018384*	2575	5095	6789	5906	5362	14,692	2.6	1.3	2.5	2.7
			*Bra031725*	1996	4409	5909	6292	5275	13,450	3.0	1.3	2.1	2.5
	AT4G27890	HSP20-like chaperones superfamily protein	*Bra040837*	77	29	232	128	199	637	3.0	8.1	5.0	3.2
	AT5G51440	HSP20-like chaperones superfamily protein	*Bra029174*	406	376	5772	1657	292	7627	14.2	15.3	4.6	26.1
	AT5G59720	Heat shock protein 18.2 (HSP18.2)	*Bra002539* *	73	259	747	206	58	5114	10.2	2.9	24.8	88.5
			*Bra006697* *	1129	779	2431	765	426	5739	2.2	3.1	7.5	13.5
			*Bra020295* *	1659	383	1167	2436	197	5383	0.7	3.0	2.2	27.3
	AT5G12020	17.6 kDa class II heat shock protein (HSP17.6II)	*Bra006137* *	52	271	2513	909	310	2036	48.6	9.3	2.2	6.6
			*Bra008920*	3065	1599	5017	1267	1426	3693	1.6	3.1	2.9	2.6

* Notable genes possibly related to the HT response in *B. rapa*.

**Table 5:** Expression of *BrHSP18.2* family genes in Chiifu and Kenshin under various temperature conditions. Expression level (fold change) was calculated based on qRT-PCR values of three replicates using *BrACT2* as a standard. Heat shock treatment was performed by increasing the temperature 5 °C every 2 h.

Gene	Line	Expression (Fold Change)								
		Heat Shock					Warming			
		22 °C	27 °C	32 °C	37 °C	42 °C	22 °C	12 °C	28 °C	45 °C
***BrHSP18.2A***	Chiifu	1.0	2.2	10.3	3220.4	4132.9	1.0	0.5	8.4	7154.1
	Kenshin	1.0	0.8	5.5	4921.5	49,617.7	1.0	0.5	106.7	34,142.0
***BrHSP18.2B***	Chiifu	1.0	0.5	8.7	74.4	357.9	1.0	1.2	1.3	241.4
	Kenshin	1.0	3.9	5.7	160.5	1738.6	1.0	1.8	42.3	31,296.8
***BrHSP18.2C***	Chiifu	1.0	1.4	13.8	140.0	646.8	1.0	1.8	7.8	7540.8
	Kenshin	1.0	2.3	4.0	84.1	2960.7	1.0	1.5	3.1	733.5

## Supplementary Materials

Supplementary materials can be found at http://www.mdpi.com/1422-0067/19/6/1727/s1.
