# Supplementary material for: Genome-Wide Identification and Characterization of Warming-Related Genes in Brassica rapa ssp. pekinensis
Source: Int J Mol Sci. 2018 Jun 11;19(6):1727. doi: 10.3390/ijms19061727 (PMC6032310; doi:10.3390/ijms19061727)
Supplement: Supplementary file 1 [file ijms-19-01727-s001.zip › Supplementary figures.docx]

**Supplementary materials**

**Figure S1.** Alternative splicing of *BrHSFA2* based on *Arabidopsis* information. **A**, Genomic organization of *BrHSFA2* gene including mini-exon and an intron. Numbers indicate size with bp. **B**, Mini-exon region in an intron showing possible alternative splicing sites (box) and stop codon (red color). **C**, Comparison of deduced amino acid sequences between *Arabidopsis* and *B. rapa* produced by alternative splicing, truncated forms of HSFA2 polypeptide.

**Figure S2.** Alignment of three BrHSP18.2 amino acid sequences. The amino acid sequences are provided as one-letter codes, and have been aligned by the introduction of gap (---) in order to illustrate homology. The sequence displayed as reverse print consists of amino acids different to one from other two sequences.

**Figure S3.** Expression of *BrSMP1* during warming and HS treatments. Real-time PCR was performed with primer set described in Table S1.

**Figure S4.** HS elements (HSEs) in *HSP18.2* promoter. Bold sequence between -97 and -53 represented eight HES modules reported by Nishizawa-Yoko et al. (2009). Boxes represented HS factor binding units in *Drosophila* (Perisic et al. 1989).
